# Supplementary material for: Identification of Adipose Tissue as a Reservoir of Macrophages after Acute Myocardial Infarction
Source: Int J Mol Sci. 2022 Sep 10;23(18):10498. doi: 10.3390/ijms231810498 (PMC9499676; doi:10.3390/ijms231810498)
Supplement: Supplementary file 1 [file ijms-23-10498-s001.zip › Supplemental Figure S1.pptx]

## Slide 1
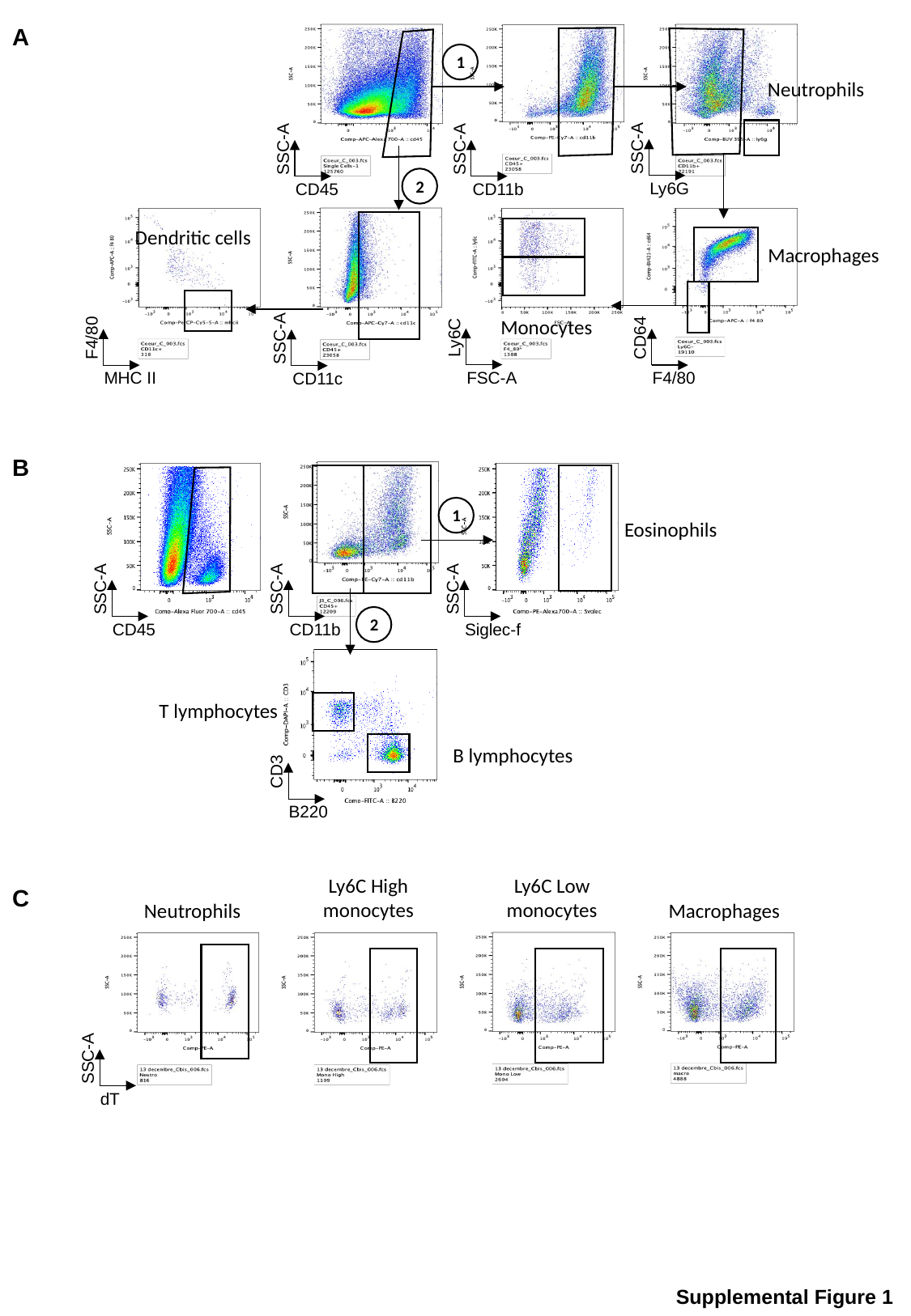

A
1
Neutrophils
SSC-A
Ly6G
SSC-A
CD45
SSC-A
CD11b
2
Dendritic cells
Macrophages
SSC-A
CD11c
CD64
F4/80
F4/80
MHC II
Ly6C
FSC-A
Monocytes
B
1
Eosinophils
SSC-A
CD45
SSC-A
CD11b
SSC-A
Siglec-f
2
T lymphocytes
B lymphocytes
CD3
B220
Ly6C High monocytes
Ly6C Low monocytes
C
Neutrophils
Macrophages
SSC-A
dT
Supplemental Figure 1
