# Supplementary material for: Identification of Adipose Tissue as a Reservoir of Macrophages after Acute Myocardial Infarction
Source: Int J Mol Sci. 2022 Sep 10;23(18):10498. doi: 10.3390/ijms231810498 (PMC9499676; doi:10.3390/ijms231810498)
Supplement: Supplementary file 1 [file ijms-23-10498-s001.zip › Supplemental Table S1.pdf]

**Supplementary Table S1** : Antibodies List

| Antibody | Clone | fluorochrome | Supplier |
|---|---|---|---|
| Anti CD45 | 30F11 | Alexa Fluor 700 ; BV510 | BD Biosciences |
| Anti CD11b | M1/70 | BV605 ; PerCP-Cy5.5 | BD Biosciences |
| Anti CD11c | HL3 | APC-Cy7 | BD Biosciences |
| Anti Ly6C | AL-21 | FITC | BD Biosciences |
| Anti Ly6G | 1A8 | PE | BD Biosciences |
| Anti-F4/80 | MCA497APC T | APC | Bio-Rad |
| Anti CD64 | X54-5/7.1 | BV421 | BioLegend |
| Anti MHCII | M5/114.15.2 | PerCP-Cy5.5 | BD Biosciences |
| Anti CD3 | 145-2C11 | BV421 | BD Biosciences |
| Anti CD4 | RM4-4 | BV650 | BD Biosciences |
| Anti CD8 | 53-6.7 | PerCP-Cy5.5 | BD Biosciences |
| Anti Ly6A/E (Sca-1) | D7 | FITC | eBiosciences |
| Anti CD45R/B220 | RA3-6B2 | BV496 | BD Biosciences |
| Anti CD117 | 2B8 | PE-Cy7 ; BV510 | BD Biosciences |
| Lineage Panel | 145-2C11 | APC | BD Bioscience |
