# Supplementary material for: Identification of Adipose Tissue as a Reservoir of Macrophages after Acute Myocardial Infarction
Source: Int J Mol Sci. 2022 Sep 10;23(18):10498. doi: 10.3390/ijms231810498 (PMC9499676; doi:10.3390/ijms231810498)
Supplement: Supplementary file 1 [file ijms-23-10498-s001.zip › Supplementary Materials rev.pdf]

Supplementary Figure Legends

Supplemental Figure S1: Gating strategy for FACS analysis of inflammatory cells

(A) Gating strategy for evaluation of myeloid cell subpopulations. Single cell tissue or blood suspensions were prepared and labeled. Size (FSC-A) and granularity (SSC-A) was assessed by flow cytometry and CD45+ leukocytes were identified, first (1) gated on CD11b and stratified by Ly6G expression. Neutrophils were identified as $CD45^{+}/CD11b^{+}/Ly6G^{+}$. $CD45^{+}/CD11b^{+}/Ly6G^{-}$ cells were also gated on CD64 and F4/80 and then further stratified by Ly6C expression. Macrophages were identified as $CD45^{+}/CD11b^{+}/Ly6G^{-}/F4/80^{+}/CD64^{+}$ and monocytes as Ly6C High or Ly6C low $CD45^{+}/CD11b^{+}/Ly6G^{-}/F4/80^{-}/CD64^{-}$ cells; second (2) gated on CD11c and stratified by F4/80 and MHCII expression. Dendritic cells were then identified as $CD45^{+}/CD11c^{+}/F4/80^{+}/MHCII^{+}$. (B) Gating strategy for evaluation of lymphocytes and eosinophil populations. Single cell tissue or blood suspensions were prepared and labeled. Size (FSC-A) and granularity (SSC-A) was assessed by flow cytometry and CD45+ leukocytes were identified, gated on CD11b and then first (1) further stratified by Siglec-f expression or CD3 and B220 expression. Eosinophils were identified as $CD45^{+}/CD11b^{+}/Siglec\text{-}f^{+}$ cells, T lymphocytes as $CD45^{+}/CD11b^{-}/CD3^{+}$ and B lymphocytes $CD45^{+}/CD11b^{-}/B220^{+}$ cells. (C) Gating strategy for evaluation of dT+ cells in neutrophil, Ly6C High and Ly6C low monocyte as well as macrophage populations, identified as described above.

Supplemental Figure S2: Characterization of AT- and BM-chimeric mice

(A) Quantification of dT^{+} and dT^{-} $Ly6C^{high}$and $Ly6C^{low}$ monocytes in the blood, BM and spleen of BM- and AT-mice, 7 days post-MI. In the stacked bars, black columns indicate the number of dT^{-} cells and colored columns the number of dT^{+} cells in BM-mice (grey) or AT-mice (blue). (n=4-7). (B) Quantification of IFNγ, TNFα, IL-12p40, CCL2, CCL3 and CCL11 levels in cardiac macrophages sorted from BM- and AT-mice, 7 days post MI (n=4-6).

Supplemental Figure S3: Effect of CD11c depletion on inflammatory chemokines in AT- and BM-chimeric mice

Quantification in the infarcted heart (A) and in the blood (B) of CCL2, CCL3 and CCL4 in BM- and AT-mice treated with or without diphtheria toxin (DT), at 7 days post MI (n=4-6). * $p<0.05$ versus PBS-treated animals.

Supplemental Figure S4: Characterization of metabolic profile and inflammatory parameters in AT- and BM-chimeric mice under HFD

Metabolic profile was investigated in chimeric mice by evaluation of (A) body weight, sub-cutaneous adipose tissue weight (n=4-5), (B) Intra peritoneal glucose tolerance test (IPGTT) and Area under curve (AUC) for IPGTT (n=4-5), and quantification of fasted glycemia (n=12-15). Quantification in the infarcted heart (C) and in the blood (D) of CCL2, CCL3 and CCL4 levels in BM and AT-mice fed with a NC or a HFD (n=4-5). * $p<0.05$; ** $p<0.01$; *** $p<0.001$ in HFD vs NC fed mice.

Supplementary Table S1

Antibody	Clone	fluorochrome	Supplier
Anti CD45	30F11	Alexa Fluor 700 ; BV510	BD Biosciences
Anti CD11b	M1/70	BV605 ; PerCP-Cy5.5	BD Biosciences
Anti CD11c	HL3	APC-Cy7	BD Biosciences
Anti Ly6C	AL-21	FITC	BD Biosciences
Anti Ly6G	1A8	PE	BD Biosciences
Anti-F4/80	MCA497APC T	APC	Bio-Rad
Anti CD64	X54-5/7.1	BV421	BioLegend
Anti MHCII	M5/114.15.2	PerCP-Cy5.5	BD Biosciences
Anti CD3	145-2C11	BV421	BD Biosciences
Anti CD4	RM4-4	BV650	BD Biosciences
Anti CD8	53-6.7	PerCP-Cy5.5	BD Biosciences
Anti Ly6A/E (Sca-1)	D7	FITC	eBiosciences
Anti CD45R/B220	RA3-6B2	BV496	BD Biosciences
Anti CD117	2B8	PE-Cy7 ; BV510	BD Biosciences
Lineage Panel	145-2C11	APC	BD Bioscience
