# Supplementary figures and images for: Identification of Adipose Tissue as a Reservoir of Macrophages after Acute Myocardial Infarction
Source: Int J Mol Sci. 2022 Sep 10;23(18):10498. doi: 10.3390/ijms231810498 (PMC9499676; doi:10.3390/ijms231810498)

## Slide 1
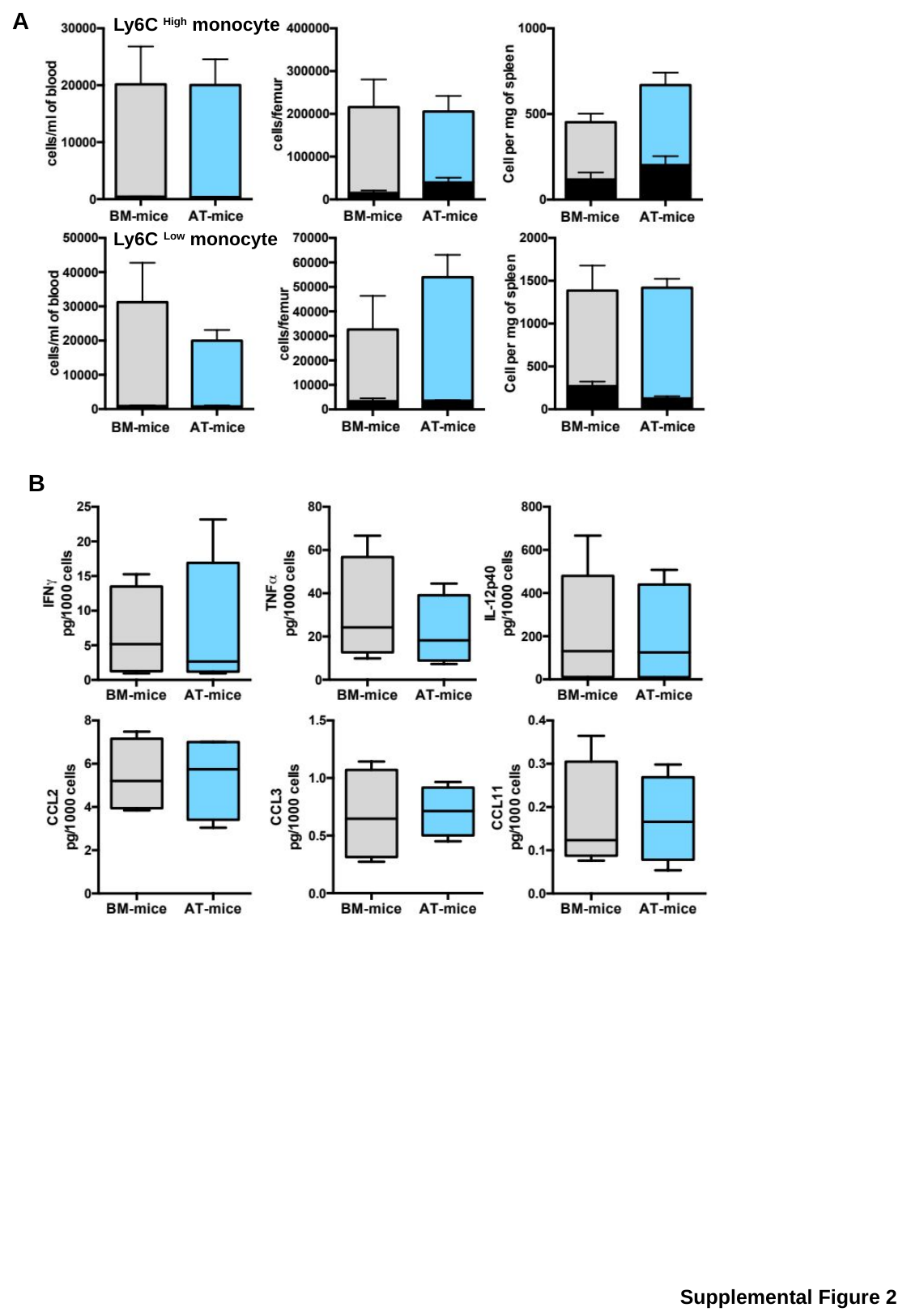

A
Ly6C High monocyte
Ly6C Low monocyte
B
Supplemental Figure 2

Supplement: Supplementary file 1 [file ijms-23-10498-s001.zip › Supplemental Figure S2.pptx]

## Slide 1
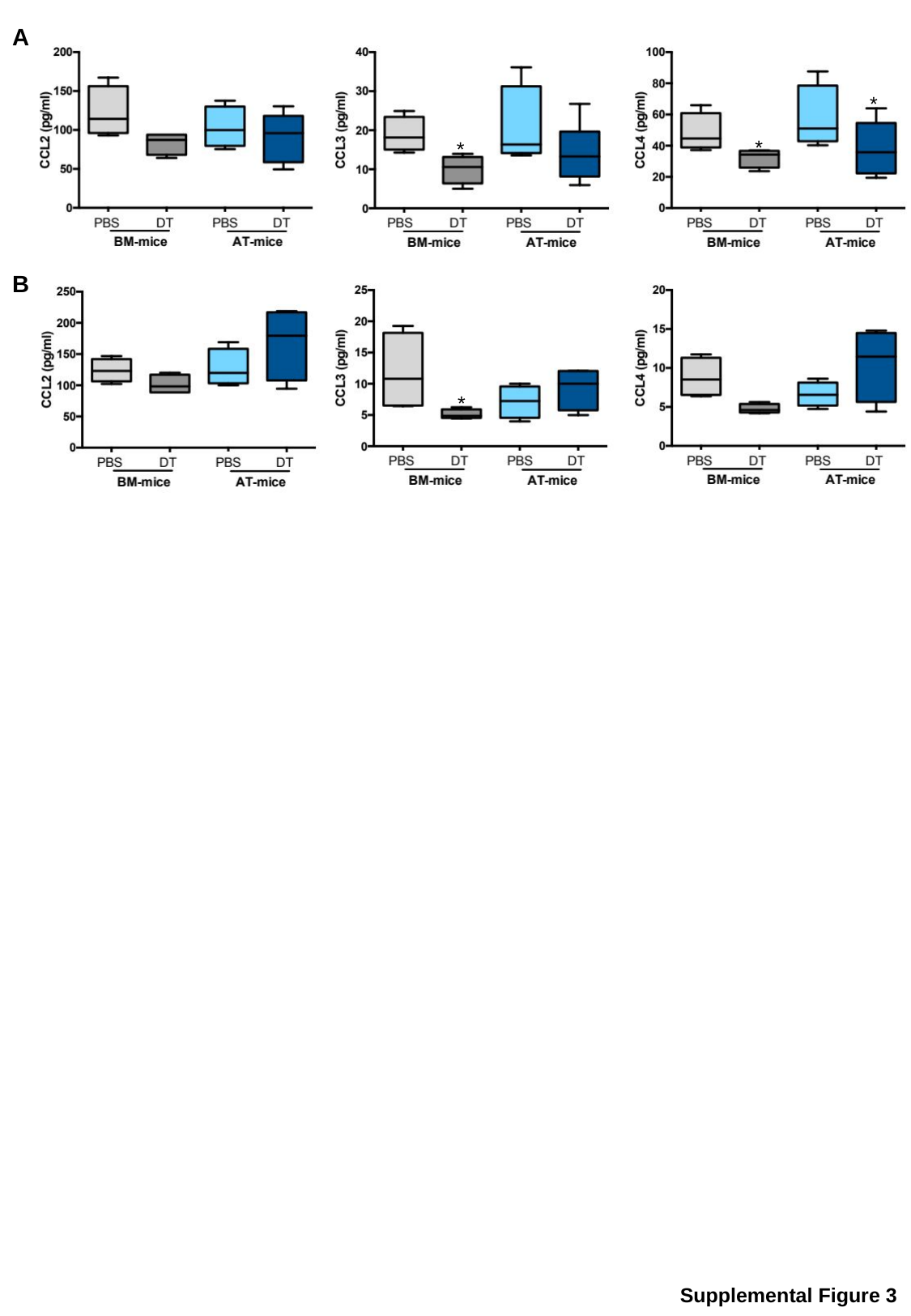

A
*
*
*
B
*
Supplemental Figure 3

Supplement: Supplementary file 1 [file ijms-23-10498-s001.zip › Supplemental Figure S3.pptx]

## Slide 1
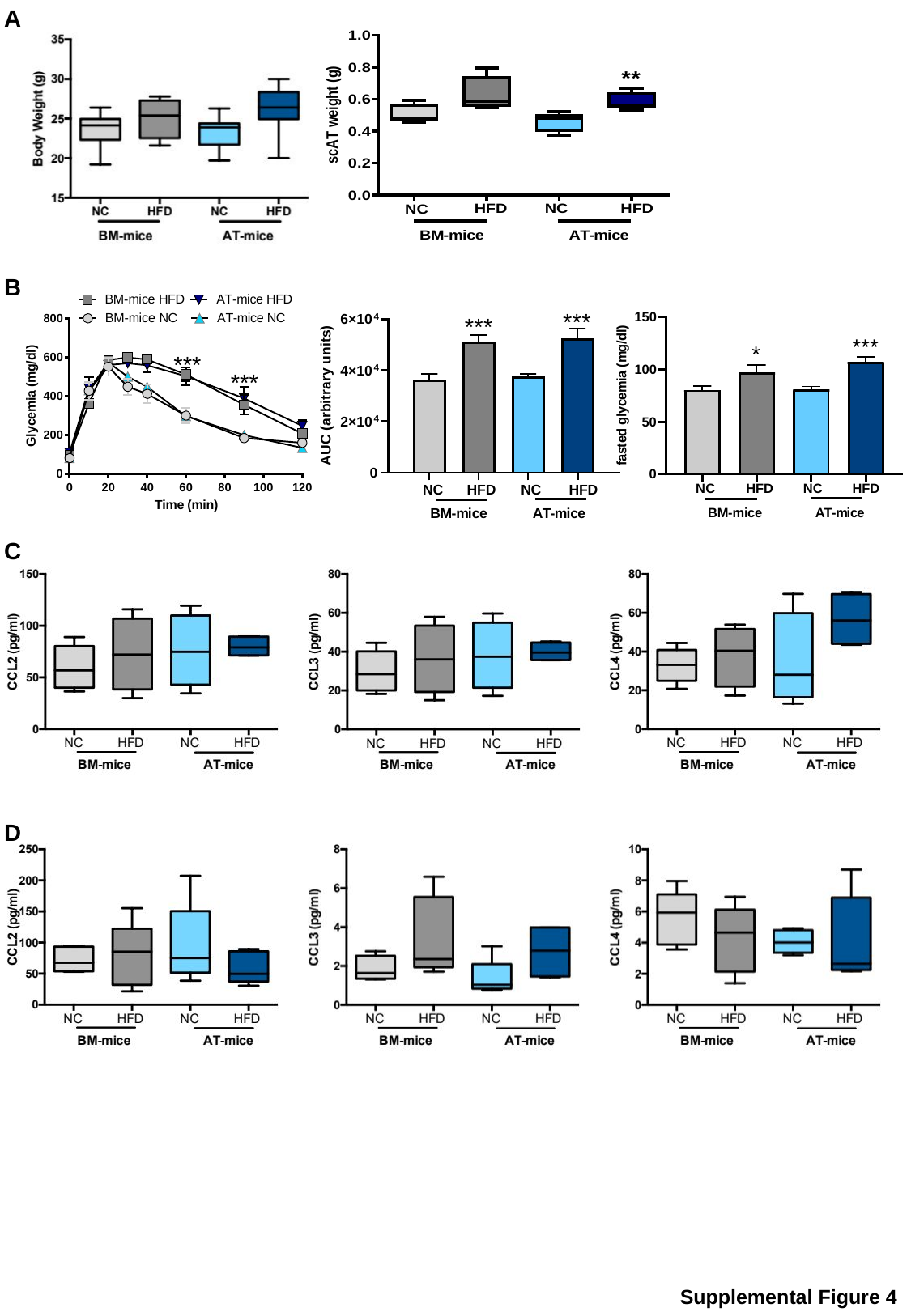

A
B
***
***
C
D
Supplemental Figure 4

Supplement: Supplementary file 1 [file ijms-23-10498-s001.zip › Supplemental Figure S4.pptx]
